# The impact of extended reality on surgery: a scoping review

**DOI:** 10.1007/s00264-022-05663-z

**Published:** 2023-01-16

**Authors:** James Zhang, Victor Lu, Vikas Khanduja

**Affiliations:** 1 School of Clinical Medicine, University of Cambridge, Cambridge, CB2 0SP UK; 2 Young Adult Hip Service, Department of Trauma and Orthopaedics, Addenbrooke’s Hospital, Cambridge University Hospital, Hills Road, Cambridge, CB2 0QQ UK

**Keywords:** Extended reality; Surgery, Scoping review, Virtual reality, Augmented reality

## Abstract

**Purpose:**

Extended reality (XR) is defined as a spectrum of technologies that range from purely virtual environments to enhanced real-world environments. In the past two decades, XR-assisted surgery has seen an increase in its use and also in research and development. This scoping review aims to map out the historical trends in these technologies and their future prospects, with an emphasis on the reported outcomes and ethical considerations on the use of these technologies.

**Methods:**

A systematic search of PubMed, Scopus, and Embase for literature related to XR-assisted surgery and telesurgery was performed using Preferred Reporting Items for Systematic reviews and Meta-Analyses extension for scoping reviews (PRISMA-ScR) guidelines. Primary studies, peer-reviewed articles that described procedures performed by surgeons on human subjects and cadavers, as well as studies describing general surgical education, were included. Non-surgical procedures, bedside procedures, veterinary procedures, procedures performed by medical students, and review articles were excluded.

Studies were classified into the following categories: impact on surgery (pre-operative planning and intra-operative navigation/guidance), impact on the patient (pain and anxiety), and impact on the surgeon (surgical training and surgeon confidence).

**Results:**

One hundred and sixty-eight studies were included for analysis. Thirty-one studies investigated the use of XR for pre-operative planning concluded that virtual reality (VR) enhanced the surgeon’s spatial awareness of important anatomical landmarks. This leads to shorter operating sessions and decreases surgical insult. Forty-nine studies explored the use of XR for intra-operative planning. They noted that augmented reality (AR) headsets highlight key landmarks, as well as important structures to avoid, which lowers the chance of accidental surgical trauma. Eleven studies investigated patients’ pain and noted that VR is able to generate a meditative state. This is beneficial for patients, as it reduces the need for analgesics. Ten studies commented on patient anxiety, suggesting that VR is unsuccessful at altering patients’ physiological parameters such as mean arterial blood pressure or cortisol levels. Sixty studies investigated surgical training whilst seven studies suggested that the use of XR-assisted technology increased surgeon confidence.

**Conclusion:**

The growth of XR-assisted surgery is driven by advances in hardware and software. Whilst augmented virtuality and mixed reality are underexplored, the use of VR is growing especially in the fields of surgical training and pre-operative planning. Real-time intra-operative guidance is key for surgical precision, which is being supplemented with AR technology. XR-assisted surgery is likely to undertake a greater role in the near future, given the effect of COVID-19 limiting physical presence and the increasing complexity of surgical procedures.

**Supplementary information:**

The online version contains supplementary material available at 10.1007/s00264-022-05663-z.

## Introduction

Modern surgery is enhanced by digital technologies which aim to improve the safety and effectiveness of procedures. These technologies include patient-specific 3D surgical planning, simulation training, navigated and robotic tools, remote assistance, and the augmentation of the operative scene with holograms [1, 2].

**Fig. 1:**
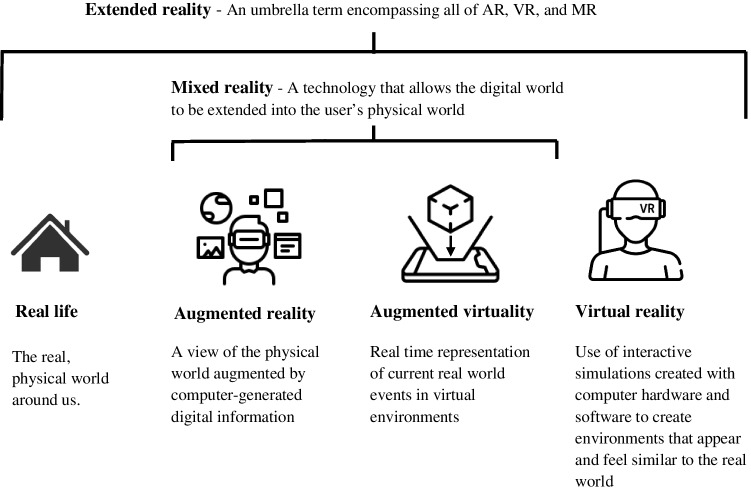
The spectrum of extended reality and its respective components

**Fig. 2:**
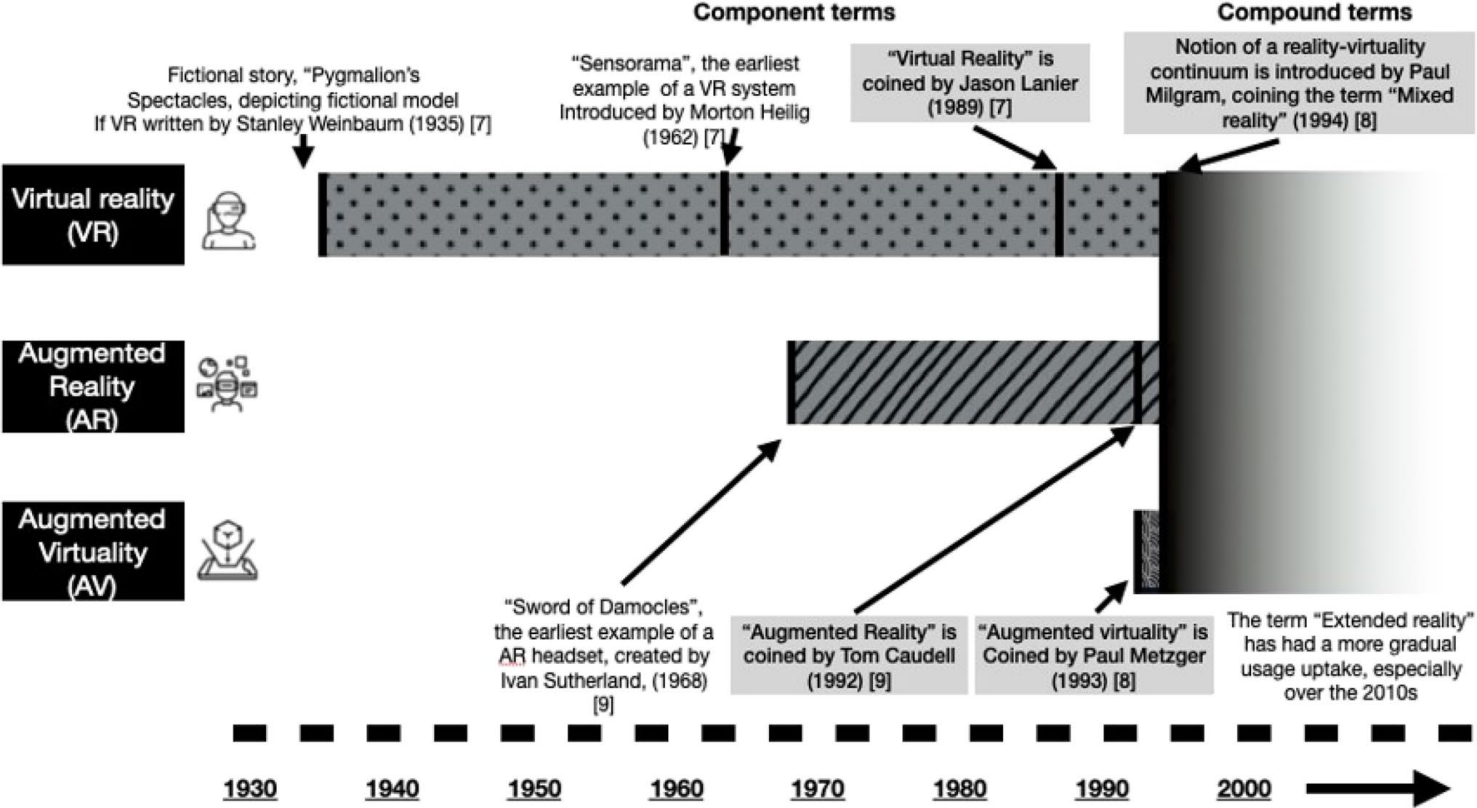
Historical Timeline of the Components of Extended Reality

Extended reality (XR) describes the display of computer-generated images (CGI) in the wearer’s field of view and is a spectrum from real life (no digital augmentation) to virtual reality (VR), whereby the user’s view of the real world is occluded (Fig. 1). VR was first described in a fictional story by Stanley Weinbaum in 1935 [3], and since then, components of XR have evolved to include augmented reality (AR) and augmented virtuality (AV) (Fig. 2). XR platforms have been utilised in many aspects of surgery: pre-operative planning, surgical training, intra-operative visualisation, and guidance, particularly for navigation around complex obscure anatomy [4]. Global restrictions in working hours and increased scrutiny of patient outcomes have improved patient safety but have reduced the exposure of surgical trainees to the volume and breadth of cases. COVID-19 has had an additional detrimental impact on surgical training, with trainees performing up to 50% fewer procedures as the primary surgeon [4], while experienced surgeons have had reduced exposure to even routine surgery and risk skill disuse [5]. In high-income countries, complex surgery is typically centralised, but in the resource-poor setting or where surgeons feel “out of practice”, XR may offer a critical layer of support and safety. For the patient, XR may have a role in improving familiarity with the upcoming procedure, facilitating better consent, and reducing peri-operative anxiety.

Given the increasing role that XR has in surgery and the growing volume of literature in this field, the aim of this scoping review was four-fold:

- Identify the type of XR most frequently used in various surgical specialities and phases of surgical intervention.
- Identify key outcome measures and trends for the use of XR in surgery.
- Determine if XR has been a promising addition to surgery, and which aspect of surgical practice has benefited the most.
- Identify opportunities and challenges for XR development and usage in the future.

## Methodology

This review was conducted based on the PRISMA extension for scoping reviews (PRISMA-ScR) guidelines [6] and Arksey and O’Malley’s 5-stage methodological framework for scoping reviews [7].

A systematic search from inception to November 25, 2020, was performed on three databases: PubMed MEDLINE, Ovid EMBASE, and Scopus. The search included variations of the following terms: surgery, outcomes, complications, augmented reality, mixed reality, virtual reality, and extended reality; the full search strategy is shown in Supplementary Table 1. Inclusion and exclusion criteria were determined using the PICOS model (population, intervention, comparison, outcome, study type) [8], as shown in Table 1. Two reviewers independently screened studies based on the inclusion and exclusion criteria. Initial screening consisted of title and abstract only, followed by a full-text review. At all stages, reasons for exclusion were documented. The senior author was contacted in case of discrepancy.

**Table 1:** PICOS inclusion and exclusion criteria for study selection

Domain	Inclusion criteria	Exclusion criteria
Population	Surgical trainees or qualified surgeons of any surgical specialty	Medical students and non-surgical personnel
Intervention	Any surgical intervention, including anaesthesia performed specifically for surgery, dentistry, radiology for surgery. Medical education for surgical procedures	Veterinary procedures. Cadaveric procedures. 3D model-assisted surgery. Rehabilitation for non-surgical procedures, bedside procedures such as phlebotomy
Comparison	N/A	N/A
Outcome	Clinical and functional outcomes, including patient satisfaction, post-operative complications, operating time, length of hospital stay	Studies where no outcome measures are directly related to efficacy of hip arthroscopy for the treatment of FAI
Study type	Primary studies written in English with full-text available	Case reports, abstracts, reviews. Studies not written in English. Animal studies

Following full-text review, included studies were classified into three categories: impact on surgery with two subgroups (pre-operative planning and intra-operative navigation/guidance), impact on the patient with two subgroups (pain and anxiety/understanding), and impact on the surgeon with two subgroups (surgical training and surgeon confidence).

## Results

**Fig. 3:**
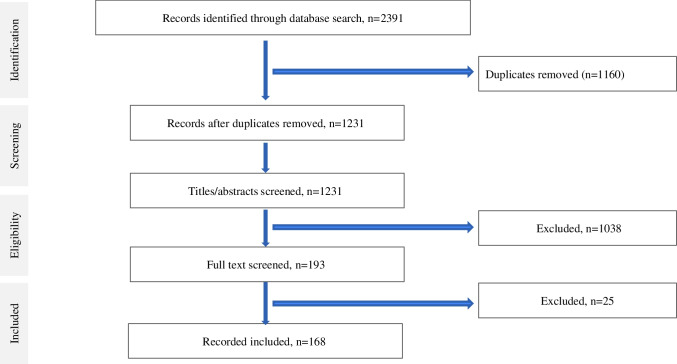
PRISMA Diagram

A total of 2391 articles were identified after the initial search from three databases. After deduplication, 1231 articles remained for the title and abstract screening, from which 238 full-text studies were reviewed. In total, 168 articles were eligible for inclusion (Supplementary Table 2). Publication dates ranged from 1998 to 2020. Figure 3 presents the PRISMA diagram.

### Impact on surgery

#### Pre-operative planning

Thirty-one papers focused on pre-operative planning. Immersive VR systems improved the surgeon’s understanding of key anatomical sites [9] and improved the localisation of key areas of lesions that need to be resected [10], which are sometimes hidden from the surgeon’s view. This led to a reduced operative time, reduced damage to the neighbouring tissue, reduced blood loss, and a shorter hospital stay [11]. Furthermore, AR is more accurate in identifying anatomical variation between patients, which can be missed on a CT scan, as AR correlates better with patient anatomy than CT scans alone [12]. VR accurately facilitated the pre-operative reconstruction of sophisticated vasculature and improved the spatial understanding of vascular anatomy, allowing safe vessel control during surgery [13]. During tumour resection, it is crucial to demarcate the boundary between the tumour and healthy tissue/vasculature; VR improves the accuracy of this process [14].

#### Intra-operative

Forty-nine papers were included in the category of intra-operative guidance and imaging for surgery. With increasing computing power and available hardware, such as Microsoft HoloLens and iPads [15], an increasing numbers of studies explored the feasibility of XR, especially AR, for intra-operative usage. Traditionally, a tri-panel display conveyed visual information to surgeons during operations; however, viewing images required looking away from the operative site. With the development of AR headsets, surgeons do not need to break their line of sight [16], with improved depth perception of fine structures [17] and motion parallax [18]. Real-time intra-operative guidance is key to defining complex structures, allowing greater surgical precision and better placement of surgical devices [12].

Intra-operative XR guidance has been well explored; thus, larger clinical trials with longer follow-up times were reported, ranging from 2 to 12 months [19–21]. Studies reporting XR usage for other surgical phases had follow-up time shorter than a patient’s hospital stay. Specialities that utilised intra-operative XR guidance the most are neurosurgery [22] and orthopaedics [23].

AR intra-operative usage involves a scan of internal structures pre-operatively using CT [24] or MRI [25]. The scan was then coupled with external marker reference points attached to the patient’s skin, providing an anchor point for hologram projections such that even with small incisions, internal structures can be visualized as an overlay image on the surface. In addition, modifications can be made that highlight “negative structures”, so that they are not accidentally incised [26], an important feature for novice surgeons. Disadvantages of this method included cables cluttering operative space [16], high additional upfront cost [27], and calibration of internal and external spaces being inconsistent [28]. Two studies reported no differences between traditional display methods and AR-assisted methods [29, 30]. One study even concluded that AR-assisted surgery is inferior in key aspects to the default [31].

### Impact on the patient

#### Pain

Eleven studies describe how XR can modulate pain for patients. XR has been used to supplement sedative and pain relief processes prior to [32], during [33], and after surgery [34]. VR has been the most prevalent form of XR used, inducing a meditative state during or after the surgery.

Usually, a VR headset, such as the Microsoft Oculus Rift, was given to the patient, with which they could try to catch imaginary creatures in the virtual realm [34], relax on a beach [35], or adventure through the Arctic tundra [36]. Outcomes of such clinical trials were compared to a control that uses conventional sedative medication. Physiological outcomes reported that less sedative is required for patients to feel at ease, increasing patient satisfaction and the desire to use it in the future [37]. This reduced patient medication, lessened side effects, and engaged the patient more in healthcare provision.

Results from the literature were heterogeneous; one study suggested no effects on pain [32], and three studies reported no change in intra-operative pain, but a reduction in sedatives was required [35, 38, 39]. Post-operatively, VR helped with pain reduction during rehabilitation and gave the patient more confidence and independence [40]. No studies have determined XR’s intra-operative effects on patients’ long-term well-being.

#### Anxiety reduction

Ten studies identified methods for anxiety reduction. Traditionally, information leaflets and conversions with the surgical team were used for patient understanding prior to surgery. XR provides a novel alternative to minimise patient anxiety, with VR being the most studied. Increasing patient understanding and diminishing anxiety ameliorates the patient experience, allowing for better cooperation.

Anxiety reduction can be split into pre-operative, intra-operative, and post-operative stages. The majority of pilot interventions were at the pre-operative stage [39, 41, 42]. Similar to pain management, meditation and exploration modules exist [41, 42]. This involved the simulation of surgical procedures or operation room tours (usually for paediatric cases) [39]. Outcomes measured include standardised scores of anxiety, such as the Hospital Anxiety and Depression Scale (HDAS), Likert type scale, visual analogue scale for anxiety (VAS-A), and modified Yale pre-operative anxiety scale (m-YPAS) [43, 44].

Some papers reported patients’ self-reported feelings as well as physiological parameters such as breathing rate, mean arterial blood pressure, heart rate, and salivary cortisol [45].

Overall, whilst VR lowered patients’ anxiety and apprehension, it did not have a significant effect on objective measurements of patients’ physiological conditions [39].

### Impact on the surgeon

#### Surgical training

Surgical training was studied in sixty papers. Surgical training has traditionally been achieved by observation and apprenticeships in operating theatres. Due to the increasing parallel duties of surgical trainees/residents, time and experience in the operating theatre come at a premium. Concern about patient safety and a reduction in working hours, coupled with the steeper learning curve of complex modern laparoscopic and arthroscopic procedures, have made surgical training an area requiring innovation [46, 47].

The most cost-effective and efficient training method has been VR; this can be done as a standalone procedure with few external inputs. AR training programs also exist, such as dentistry, whereby the AR headset interacts with a 3D model, with the trainee using live tools monitored electronically. Specialities with frequent VR usage were variations in laparoscopic procedures [48] and orthopaedic procedures [49].

The time frame of VR training sessions is usually a few months. Surgical simulators can be divided into two groups: basic isolated skills trainers versus specific procedural task trainers [50] and low-fidelity versus high-fidelity simulators [51]. Outcome measures seen in the literature included an overall judgement of skills by experts in the field and measurements of objective performance using procedure-specific scores [52]. Quantitative metrics were commonplace, such as time of surgery [53], complication rate [54], tool path length/tissue protection [55], degree of hand/head movement [56], and self-reported feelings [57].

Two studies suggested that these training programs increase surgical trainees’ competence when they perform their first surgery on a real patient. One study reported on the total cost of developing their own simulators, estimated at $4000–$8000 [58].

The literature included four main ways to assess training programs: construct validity (the ability to measure what it claims to be measuring), face validity (whether the program is suitable for its aims), content validity (the degree to which it is fully representative of what it aims to measure), and concurrent validity (how well a test performs compared to its well-established predecessors). Construct validity was assessed by comparing objective outcome metrics between a spectrum of surgeons on different sections of the learning curve [59]. Outcomes that determine their score were the time of surgery, tool path length, and surgical errors. A statistically significant difference indicates this program can stratify surgeons according to skill level.

Other studies attempted to convert the program into a test of skills, by either using a large number of surgeons’ performance in the program as a benchmarking distribution [56], using experts’ program performance as the benchmark [60], or correlating program performance with a real surgical performance by the same participant [61]. The latter may have ethical implications, since non-experienced surgeons are allowed to independently operate on patients in order to calibrate the scale’s extreme ends. Inter-procedural calibration was also achieved by correlating scores of different procedures in the same field [59]. Face validity was demonstrated by obtaining surgeons’ views with questionnaires on a range of aspects after they have used the program [62]. The majority of studies reported their training and assessment programs to be valid for their purposes.

#### Surgeon confidence

Seven papers documented surgeon confidence. The majority focused on VR-assisted “warm up” tasks prior to surgery [63], as well as pre-operative planning [64]. There was a significant increase in subjective confidence and improved objective parameters in the “warmed up” group, although the effects are diminished if the VR task was not done immediately before surgery [65]. VR’s beneficial effects were augmented for senior surgeons, for more complex procedures, and when there was real-time feedback during the “warm up” from more experienced surgeons.

## Discussion

This scoping review showed the wide range of specialities and roles in which XR has been deployed to improve surgery. There has been an exponential increase in studies demonstrating the development and deployment of XR—in particular, VR and AR—for surgical training, pre-operative planning, intra-operative surgical performance, and to improve patients’ experience of surgery. This is likely driven by advances in the capability and affordability of XR headsets in the last decade and the increasing acceptance of these technologies by surgeons and patients.

**Fig. 4:**
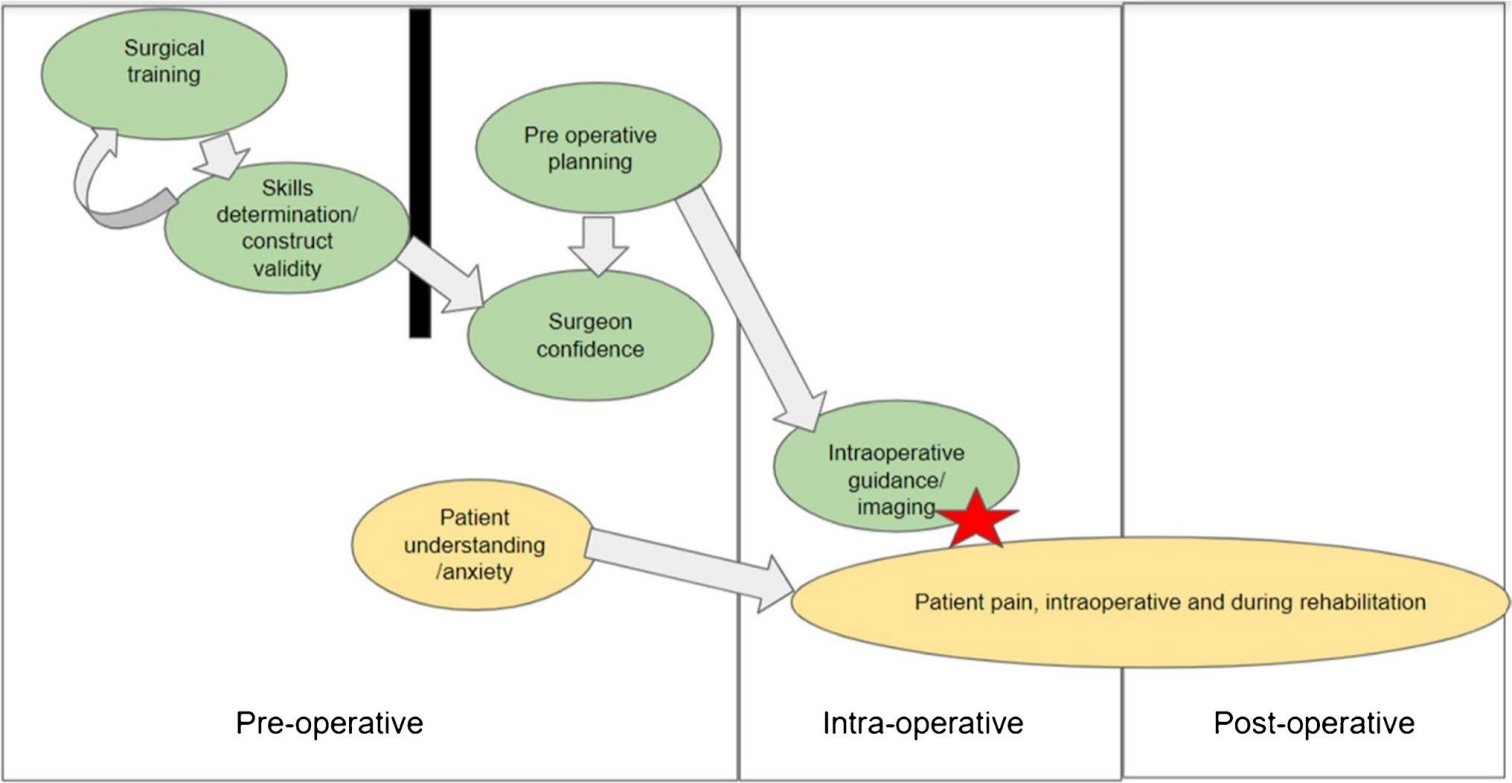
Flowchart showing synchronisation of technologies at different stages

**Fig. 5:**
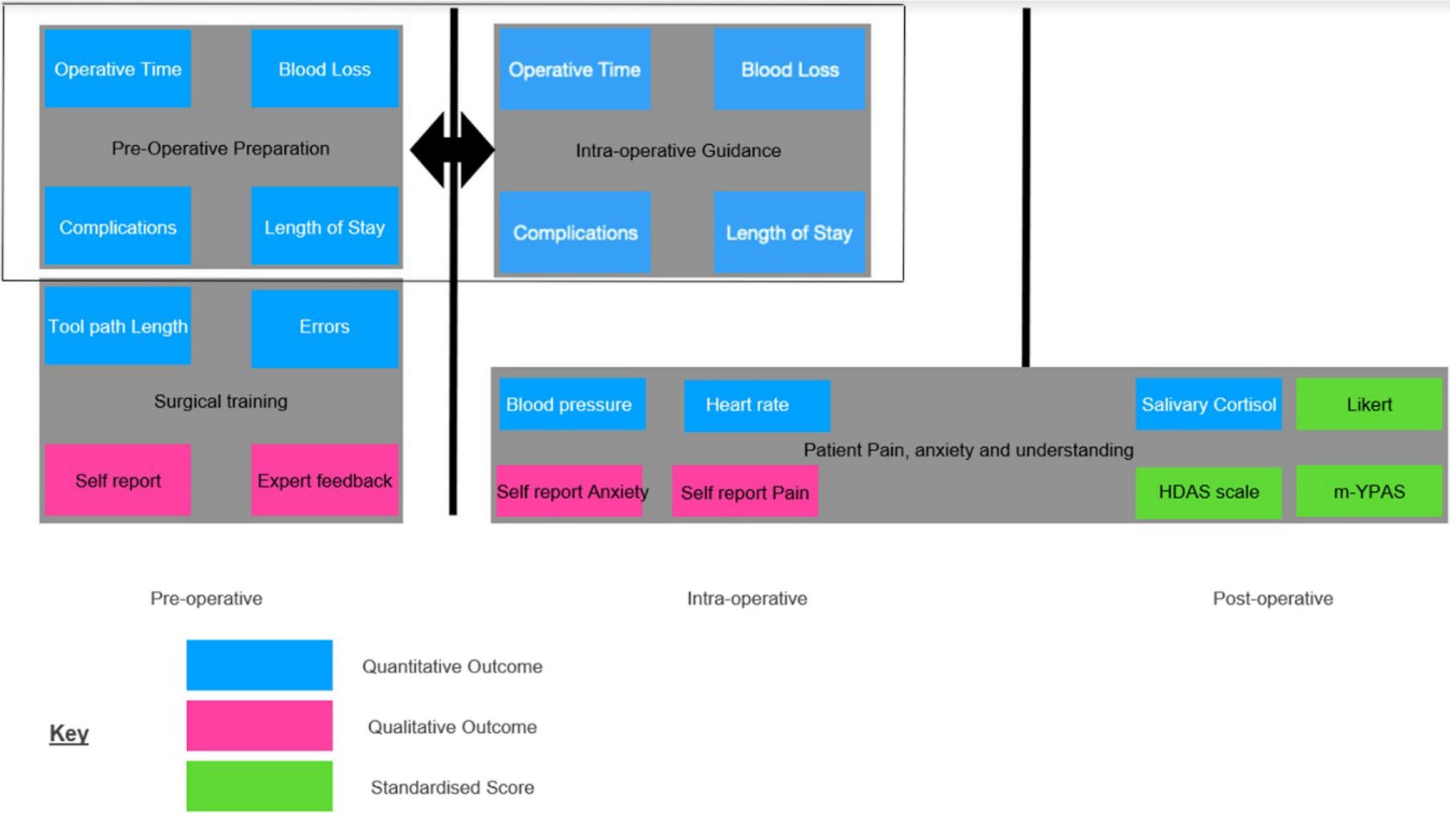
Flowchart summarising outcome measures reported

The main themes identified are pre-operative planning, intra-operative guidance/imaging, and surgical training. Regarding the type of XR used, surgical training was mostly assisted by VR and intra-operative guidance by AR. The other sections of the XR spectrum, namely augmented virtuality and mixed reality, are relatively underexplored. In augmented virtuality, a real-world object is directly added into a simulated environment, and whilst it is able to isolate the issue in a system of simulated environments, often the entire patient is taken into account when planning procedures. Thus, often, augmented virtuality will be replaced by either augmented reality or virtual reality. The definition of mixed reality is still ambiguous in the literature and was mentioned once in one study [66]. Figure 4 summarises how XR has been integrated into different phases of surgery, organised by the impact on the surgeon and the surgical procedure, or the patient. Figure 5 depicts the various outcome measures reported in the literature on XR-assisted surgery.

The highest quality evidence for XR-assisted surgery was identified in relation to surgical training. Having surgeons train in simulators that allow them to virtually practice with the same tools which they later use in real surgery diminished unfamiliarity, removed part of the learning curve from the real operating room, and enabled the measurement of acceptable levels of competency. This could streamline the selection, training, and certification processes.

XR (specifically AR) has been developed more for intra-operative assistance in open procedures rather than endoscopic surgery. This may change in the coming decade, as several studies have demonstrated that 3D displays may result in better accuracy, faster surgery, and fewer errors in endoscopic surgery [67–69]. Thus far, VR surgical training had been most utilised for endoscopic procedures, and in particular for procedures that have longer learning curves such as hip arthroscopy and mastoid surgery [56, 70, 71]. However, a number of recent trials have demonstrated the positive impact of immersive VR [49, 52, 58, 72–76] and AR for surgical training for open procedures [59, 77]. Again, the balance between XR for endoscopic versus open surgery simulation is likely to shift in the coming decade with the advent of affordable and effective iVR training platforms.

With the exception of a number of studies evaluating XR-assisted intra-operative guidance, there was a paucity of studies directly comparing these technologies to a control group [29, 31, 78, 79]. The few studies that undertook direct comparisons often showed insignificant differences. This could be due to publication bias, since the results of smaller-scale studies investigating novel devices are likely to be shared, whereas real-world testing of novel technologies is complex, and many factors other than the use of the XR intervention can impact outcomes. Instead, these studies demonstrate the safety and equivalence of the technologies [16, 17, 26, 80–82] and large studies and long-term data are required to adequately examine their impact.

The follow-up time in most studies was short, with most outcomes reported either intra- or immediately post-operatively. However, some surgeon-oriented studies in this area investigated long-term career progression and outcomes of XR-assisted training and assessment [83].

Since the start of the twenty-first century, the literature has shown two major periods of advancement: the 2000s were focused on VR for endoscopic simulation, telesurgery trials, and proof of concept studies in stereoscopic displays, holograms, and other guises of AR. The development, validation, and deployment of XR have dominated the literature since 2010, with particular advancements in AR and immersive VR headsets. We did not identify any data investigating patients’ views on XR-assisted surgery. Instead, there has been a greater focus on the cost, reliability, and feasibility of these technologies to establish their place in surgery, when compared to studies published in the early 2000s [84]. The patient viewpoint is of particular relevance due to increased international scrutiny on how patient’s data is used and how their privacy is protected and requires further research.

We propose three main areas of research that future studies could explore:

1. Conducting large-scale studies with adequately designed control arms will provide more statistical power to any differences in outcomes between novel technologies and the status quo. With the exception of intra-operative XR assistance, these are lacking in the literature.
2. Patient’s views regarding the new technologies should be collated and reported. Currently, only those directly impacting the patient, such as pain/anxiety reduction, are qualitatively analysed. Patients should have the opportunity to express their views on all aspects of their surgical journey.
3. Longer follow-up times are needed in studies to investigate the long-term career impacts on surgeons and the long-term impacts on patients’ quality of life.

## Limitations

A scoping review is a novel clinical research method for mapping out the literature in a novel and rapidly advancing research field like XR-assisted surgery, in order to suggest gaps in the literature for future primary studies and systematic reviews. The inclusion criteria were limited to primary studies involving surgical personnel, meaning that novel theoretical papers were missed, as well as trials involving the general public. Nevertheless, these limitations were necessary to avoid commenting on hypotheticals that may not translate to clinical practice. Similarly, the exclusion of grey literature and non-English papers was a decision made to focus on well-established studies published in international journals. In such a rapidly growing field, some novel research may have occurred since the time we performed our literature search; hence, relevant studies may have been missed in the intervening time.

## Conclusions

This scoping review provides an overview of XR-assisted surgery, detailing its history and developments in the past two decades, with an emphasis on surgical specialties, outcome measures, and the utility of the technologies for the surgeon and patient. Currently, the most studied areas of XR-assisted surgery are surgical training, pre-operative preparation, and intra-operative guidance and imaging. Training and pre-operative preparation are mainly achieved by virtual reality, whereas intra-operative guidance is supplemented mainly with augmented reality.

In the past decade, XR-assisted surgery has seen significant growth, fuelled by technological advances in hardware and software. As XR is now affordable, usable, acceptable, and increasingly well-validated, we recommend future studies focus on improving methodological rigour and longer follow-up, with a new focus on understanding patients’ views of these novel technologies.

## Supplementary information

Below is the link to the electronic supplementary material.

Supplementary file1 (DOCX 14 KB)

Supplementary file2 (DOCX 44 KB)

## Data Availability

The authors confirm that the data supporting the findings of this study are available within the article [and/or] its supplementary materials.
