# Supplementary material for: The impact of extended reality on surgery: a scoping review
Source: Int Orthop. 2023 Jan 16;47(3):611–21. doi: 10.1007/s00264-022-05663-z (PMC9841146; doi:10.1007/s00264-022-05663-z)
Supplement: Supplementary file 1 — Supplementary file1 (DOCX 14 KB) [file 264_2022_5663_MOESM1_ESM.docx]

| **Supplementary table 1**: Detailed search strategy | | | |
| --- | --- | --- | --- |
| **Extended Reality variations:** | **Surgery variations:** | **Outcome variations:** |  |
| 1) Augmented reality | 7) surg* | 13) outcome | 20) 6 AND 12 AND 19 |
| 2) Mixed reality | 8) resect* | 14) complication |  |
| 3) Virtual reality | 9) incision | 15) consequence |  |
| 4) Extended reality | 10) operation | 16) effect |  |
| 5) Augmented virtuality | 11) operating theatre | 17) result |  |
| 6) 1 OR 2 OR 3 OR 4 OR 5 | 12) 7 OR 8 OR 9 OR 10 OR 11 | 18) end result |  |
|  |  | 19) 13 OR 14 OR 15 OR 16 OR 17 OR 18 |  |
| Databases searched: Ovid EMBASE, PubMed MEDLINE, Web of Science.  Databases were searched from inception to November 25^th^ 2020. No filters or limits were used. | | | |
