# Supplementary material for: The impact of extended reality on surgery: a scoping review
Source: Int Orthop. 2023 Jan 16;47(3):611–21. doi: 10.1007/s00264-022-05663-z (PMC9841146; doi:10.1007/s00264-022-05663-z)
Supplement: Supplementary file 2 — Supplementary file2 (DOCX 44 KB) [file 264_2022_5663_MOESM2_ESM.docx]

**Supplementary Table 2**: 168 studies were included for analysis

| **Pre-Operative Planning** |
| --- |
| Porpiglia, F., Checcucci, E., Amparore, D., Piramide, F., Volpi, G., Granato, S., ... & Mottrie, A. Three-dimensional augmented reality robot-assisted partial nephrectomy in case of complex tumours (PADUA≥ 10): a new intraoperative tool overcoming the ultrasound guidance. European urology,(2020). 78(2), 229-238. |
| Yan EG, Rennert RC, Levy DM, Levy ML. Three-Dimensional Modeling of Complex Pediatric Intracranial Aneurysmal Malformations With a Virtual Reality System [published online ahead of print, 2020 Sep 2]. Simul Healthc. 2020;10.1097/SIH.0000000000000498. doi:10.1097/SIH.0000000000000498 |
| WANG, S. S., ZHANG, H. J., JING, J. J., WEI, L. F., & WANG, R. M.. Application of virtual reality techniques in preoperative surgical planning for intracranial anterior circulation aneurysms. Medical Journal of Chinese People's Liberation Army, (2012) 37(5), 393-397. |
| Gacto-Sánchez P, Sicilia-Castro D, Gómez-Cía T, et al. Use of a three-dimensional virtual reality model for preoperative imaging in DIEP flap breast reconstruction. J Surg Res. 2010;162(1):140-147. doi:10.1016/j.jss.2009.01.025 |
| Shirk JD, Thiel DD, Wallen EM, et al. Effect of 3-Dimensional Virtual Reality Models for Surgical Planning of Robotic-Assisted Partial Nephrectomy on Surgical Outcomes: A Randomized Clinical Trial. JAMA Netw Open. 2019;2(9):e1911598. Published 2019 Sep 4. doi:10.1001/jamanetworkopen.2019.11598 |
| Shirk JD, Kwan L, Saigal C. The Use of 3-Dimensional, Virtual Reality Models for Surgical Planning of Robotic Partial Nephrectomy. Urology. 2019;125:92-97. doi:10.1016/j.urology.2018.12.026 |
| Ma S, Chen S, Hu Y, et al. Zhonghua Yi Xue Za Zhi. 2014;94(45):3562-3566. |
| Guerriero L, Quero G, Diana M, et al. Virtual Reality Exploration and Planning for Precision Colorectal Surgery. Dis Colon Rectum. 2018;61(6):719-723. doi:10.1097/DCR.0000000000001077 |
| Lima M, Reinberg O, Ruggeri G, et al. 3D virtual rendering before laparoscopic partial splenectomy in children. J Pediatr Surg. 2013;48(8):1784-1788. doi:10.1016/j.jpedsurg.2013.06.011 |
| Gao, W., Bi, Y., Ye, M., Wu, C., Wang, Z., & Yu, Z.. Application of virtual reality technology in the operation of brain tumors in central cortex and adjacent areas. Chinese Journal of Neurosurgery, (2020) 36, 264-268. |
| Kockro RA, Killeen T, Ayyad A, et al. Aneurysm Surgery with Preoperative Three-Dimensional Planning in a Virtual Reality Environment: Technique and Outcome Analysis. World Neurosurg. 2016;96:489-499. doi:10.1016/j.wneu.2016.08.124 |
| Application of virtual reality technology in the operation of brain tumors in central cortex and adjacent areas |
| Tianming, Q., Jinsong, W., Yi, Z., & Liangfu, Z. . PRESURGICAL PLANNING FOR CEREBRAL GLIOMA WITH PYRAMIDAL TRACT INVOLVEMENT IN A STEREOSCOPIC DTI-BASED VIRTUAL REALITY ENVIRONMENT. In NEURO-ONCOLOGY (2009, December) (Vol. 11, No. 6, pp. 881-881). JOURNALS DEPT, 2001 EVANS RD, CARY, NC 27513 USA: OXFORD UNIV PRESS INC. |
| Chessa, F., Schiavina, R., Bianchi, L., Marcelli, E., Diciotti, S., Lodi, S., ... & Brunocilla, E.. Three dimensional model of the prostate and augmented reality robot assisted radical prostatectomy: A randomized controlled study to evaluate intraoperative and pathologic outcomes. European Urology Open Science, (2020) 19, e1774-e1775. |
| Zawy Alsofy S, Sakellaropoulou I, Stroop R. Evaluation of Surgical Approaches for Tumor Resection in the Deep Infratentorial Region and Impact of Virtual Reality Technique for the Surgical Planning and Strategy. J Craniofac Surg. 2020;31(7):1865-1869. doi:10.1097/SCS.0000000000006525 |
| Peloso, A., Vajana, J. D. T., Fiorina, I., Franchi, E., Barbieri, L., Quaretti, P., ... & Maestri, M.. USE OF PREOPERATIVE PLANNING IN HEPATIC SURGERY. A TOOL TO IDENTIFY A PATIENTSPECIFIC APPROACH: 526. The Official Journal of The International Hepato Pancreato Biliary Association, (2012) 14, 542-543. |
| Zawwar, A., Feeney, J., O'Neill, A., & Neary, P. . Assessment of virtual reality colonoscopy in the surgical planning of stage IV penetrating colorectal endometriosis. In IRISH JOURNAL OF MEDICAL SCIENCE (2016, March)(Vol. 185, pp. S121-S121). 236 GRAYS INN RD, 6TH FLOOR, LONDON WC1X 8HL, ENGLAND: SPRINGER LONDON LTD. |
| Alexander C, Loeb AE, Fotouhi J, Navab N, Armand M, Khanuja HS. Augmented Reality for Acetabular Component Placement in Direct Anterior Total Hip Arthroplasty. J Arthroplasty. 2020;35(6):1636-1641.e3. doi:10.1016/j.arth.2020.01.025 |
| D’Urso, A., Agnus, V., Barberio, M. et al. Computer-assisted quantification and visualization of bowel perfusion using fluorescence-based enhanced reality in left-sided colonic resections. Surg Endosc (2020). https://doi.org/10.1007/s00464-020-07922-9 |
| Ogawa H, Hasegawa S, Tsukada S, Matsubara M. A Pilot Study of Augmented Reality Technology Applied to the Acetabular Cup Placement During Total Hip Arthroplasty. J Arthroplasty. 2018;33(6):1833-1837. doi:10.1016/j.arth.2018.01.067 |
| Sugimoto M, Yasuda H, Koda K, et al. Carbon dioxide-enhanced virtual MDCT cholangiopancreatography. J Hepatobiliary Pancreat Sci. 2010;17(5):601-610. doi:10.1007/s00534-009-0201-8 |
| Bourdel N, Collins T, Pizarro D, et al. Use of augmented reality in laparoscopic gynecology to visualize myomas. Fertil Steril. 2017;107(3):737-739. doi:10.1016/j.fertnstert.2016.12.016 |
| Shen F, Chen B, Guo Q, Qi Y, Shen Y. Augmented reality patient-specific reconstruction plate design for pelvic and acetabular fracture surgery. Int J Comput Assist Radiol Surg. 2013;8(2):169-179. doi:10.1007/s11548-012-0775-5 |
| Gómez-Cía T, Gacto-Sánchez P, Sicilia D, et al. The virtual reality tool VirSSPA in planning DIEP microsurgical breast reconstruction. Int J Comput Assist Radiol Surg. 2009;4(4):375-382. doi:10.1007/s11548-009-0311-4 |
| Qiu TM, Zhang Y, Wu JS, et al. Virtual reality presurgical planning for cerebral gliomas adjacent to motor pathways in an integrated 3-D stereoscopic visualization of structural MRI and DTI tractography. Acta Neurochir (Wien). 2010;152(11):1847-1857. doi:10.1007/s00701-010-0739-x |
| Tang HL, Sun HP, Gong Y, et al. Preoperative surgical planning for intracranial meningioma resection by virtual reality. Chin Med J (Engl). 2012;125(11):2057-2061. |
| Tel A, Bagatto D, Tuniz F, et al. The evolution of craniofacial resection: A new workflow for virtual planning in complex craniofacial procedures. J Craniomaxillofac Surg. 2019;47(9):1475-1483. doi:10.1016/j.jcms.2019.06.016 |
| Lyuksemburg, V., Abou-Hanna, J., Marshall, J. S., Stroumpi, E., Keller, S. M. P., Bramlet, M. T., & Orcutt, S. T. . Virtual Reality for Preoperative Planning in Complex Surgical Oncology: The Initial Experience. Journal of the American College of Surgeons,(2020) 231(4), e215. |
| van Duren BH, Sugand K, Wescott R, Carrington R, Hart A. Augmented reality fluoroscopy simulation of the guide-wire insertion in DHS surgery: A proof of concept study. Med Eng Phys. 2018;55:52-59. doi:10.1016/j.medengphy.2018.02.007 |
| Zhu M, Chai G, Lin L, et al. Effectiveness of a Novel Augmented Reality-Based Navigation System in Treatment of Orbital Hypertelorism. Ann Plast Surg. 2016;77(6):662-668. doi:10.1097/SAP.0000000000000644 |
| Zawy Alsofy S, Stroop R, Fusek I, et al. Virtual Reality-Based Evaluation of Surgical Planning and Outcome of Monosegmental, Unilateral Cervical Foraminal Stenosis. World Neurosurg. 2019;129:e857-e865. doi:10.1016/j.wneu.2019.06.057 |
| **Intra-Operative Guidance** |
| Alexander C, Loeb AE, Fotouhi J, Navab N, Armand M, Khanuja HS. Augmented Reality for Acetabular Component Placement in Direct Anterior Total Hip Arthroplasty. J Arthroplasty. 2020;35(6):1636-1641.e3. doi:10.1016/j.arth.2020.01.025 |
| Ogawa H, Hasegawa S, Tsukada S, Matsubara M. A Pilot Study of Augmented Reality Technology Applied to the Acetabular Cup Placement During Total Hip Arthroplasty. J Arthroplasty. 2018;33(6):1833-1837. doi:10.1016/j.arth.2018.01.067 |
| Auloge P, Cazzato RL, Ramamurthy N, et al. Augmented reality and artificial intelligence-based navigation during percutaneous vertebroplasty: a pilot randomised clinical trial. Eur Spine J. 2020;29(7):1580-1589. doi:10.1007/s00586-019-06054-6 |
| Hu MH, Chiang CC, Wang ML, Wu NY, Lee PY. Clinical feasibility of the augmented reality computer-assisted spine surgery system for percutaneous vertebroplasty. Eur Spine J. 2020;29(7):1590-1596. doi:10.1007/s00586-020-06417-4 |
| Liu H, Wu J, Tang Y, et al. Percutaneous placement of lumbar pedicle screws via intraoperative CT image-based augmented reality-guided technology [published online ahead of print, 2019 Dec 20]. J Neurosurg Spine. 2019;1-6. doi:10.3171/2019.10.SPINE19969 |
| Jean WC, Huang MC, Felbaum DR. Optimization of skull base exposure using navigation-integrated, virtual reality templates. J Clin Neurosci. 2020;80:125-130. doi:10.1016/j.jocn.2020.08.018 |
| Wei P, Yao Q, Xu Y, Zhang H, Gu Y, Wang L. Percutaneous kyphoplasty assisted with/without mixed reality technology in treatment of OVCF with IVC: a prospective study. J Orthop Surg Res. 2019;14(1):255. Published 2019 Aug 8. doi:10.1186/s13018-019-1303-x |
| Edström E, Burström G, Persson O, et al. Does Augmented Reality Navigation Increase Pedicle Screw Density Compared to Free-Hand Technique in Deformity Surgery? Single Surgeon Case Series of 44 Patients. Spine (Phila Pa 1976). 2020;45(17):E1085-E1090. doi:10.1097/BRS.0000000000003518 |
| Ma L, Zhao Z, Zhang B, et al. Three-dimensional augmented reality surgical navigation with hybrid optical and electromagnetic tracking for distal intramedullary nail interlocking. Int J Med Robot. 2018;14(4):e1909. doi:10.1002/rcs.1909 |
| Pietruski P, Majak M, Świątek-Najwer E, et al. Supporting fibula free flap harvest with augmented reality: A proof-of-concept study. Laryngoscope. 2020;130(5):1173-1179. doi:10.1002/lary.28090 |
| Puliatti, S., Sighinolfi, M. C., Rocco, B., Patel, V., Francesco, P., Micali, S., ... & Bianchi, G.. First live case of augmented reality robot-assisted radical prostatectomy from 3D magnetic resonance imaging reconstruction integrated with PRECE model (Predicting Extracapsular extension of prostate cancer). Urology Video Journal, (2019) 1, 100002. |
| Tang R, Ma L, Xiang C, et al. Augmented reality navigation in open surgery for hilar cholangiocarcinoma resection with hemihepatectomy using video-based in situ three-dimensional anatomical modeling: A case report. Medicine (Baltimore). 2017;96(37):e8083. doi:10.1097/MD.0000000000008083 |
| Marcus HJ, Pratt P, Hughes-Hallett A, et al. Comparative effectiveness and safety of image guidance systems in surgery: a preclinical randomised study. Lancet. 2015;385 Suppl 1:S64. doi:10.1016/S0140-6736(15)60379-8 |
| Gan A, Cohen A, Tan L. Augmented Reality-Assisted Percutaneous Dilatational Tracheostomy in Critically Ill Patients With Chronic Respiratory Disease. J Intensive Care Med. 2019;34(2):153-155. doi:10.1177/0885066618791952 |
| Battaglia S, Badiali G, Cercenelli L, et al. Combination of CAD/CAM and Augmented Reality in Free Fibula Bone Harvest. Plast Reconstr Surg Glob Open. 2019;7(11):e2510. Published 2019 Nov 22. doi:10.1097/GOX.0000000000002510 |
| Zhu M, Liu F, Chai G, et al. A novel augmented reality system for displaying inferior alveolar nerve bundles in maxillofacial surgery. Sci Rep. 2017;7:42365. Published 2017 Feb 15. doi:10.1038/srep42365 |
| Citardi MJ, Agbetoba A, Bigcas JL, Luong A. Augmented reality for endoscopic sinus surgery with surgical navigation: a cadaver study. Int Forum Allergy Rhinol. 2016;6(5):523-528. doi:10.1002/alr.21702 |
| Ma L, Zhao Z, Chen F, Zhang B, Fu L, Liao H. Augmented reality surgical navigation with ultrasound-assisted registration for pedicle screw placement: a pilot study. Int J Comput Assist Radiol Surg. 2017;12(12):2205-2215. doi:10.1007/s11548-017-1652-z |
| Schiavina, R., Angiolini, A., Bianchi, L., Barbaresi, U., Porreca, A., Chessa, F., ... & Brunocilla, E.. Imaging guided surgery with augmented reality for robotic partial nephrectomy. European Urology Open Science, (2020) 19, e2412. |
| Ieiri S, Uemura M, Konishi K, et al. Augmented reality navigation system for laparoscopic splenectomy in children based on preoperative CT image using optical tracking device. Pediatr Surg Int. 2012;28(4):341-346. doi:10.1007/s00383-011-3034-x |
| Souzaki R, Ieiri S, Uemura M, et al. An augmented reality navigation system for pediatric oncologic surgery based on preoperative CT and MRI images. J Pediatr Surg. 2013;48(12):2479-2483. doi:10.1016/j.jpedsurg.2013.08.025 |
| Liu WP, Azizian M, Sorger J, et al. Cadaveric feasibility study of da Vinci Si-assisted cochlear implant with augmented visual navigation for otologic surgery. JAMA Otolaryngol Head Neck Surg. 2014;140(3):208-214. doi:10.1001/jamaoto.2013.6443 |
| Witowski J, Darocha S, Kownacki Ł, et al. Augmented reality and three-dimensional printing in percutaneous interventions on pulmonary arteries. Quant Imaging Med Surg. 2019;9(1):23-29. doi:10.21037/qims.2018.09.08 |
| Tomikawa M, Hong J, Shiotani S, et al. Real-time 3-dimensional virtual reality navigation system with open MRI for breast-conserving surgery. J Am Coll Surg. 2010;210(6):927-933. doi:10.1016/j.jamcollsurg.2010.01.032 |
| Schiavina R, Bianchi L, Lodi S, et al. Real-time Augmented Reality Three-dimensional Guided Robotic Radical Prostatectomy: Preliminary Experience and Evaluation of the Impact on Surgical Planning [published online ahead of print, 2020 Aug 31]. Eur Urol Focus. 2020;S2405-4569(20)30217-0. doi:10.1016/j.euf.2020.08.004 |
| Jean WC, Felbaum DR. The Use of Augmented Reality to Improve Safety of Anterior Petrosectomy: Two-Dimensional Operative Video. World Neurosurg. 2021;146:162. doi:10.1016/j.wneu.2020.11.054 |
| Pellegrino G, Mangano C, Mangano R, Ferri A, Taraschi V, Marchetti C. Augmented reality for dental implantology: a pilot clinical report of two cases. BMC Oral Health. 2019;19(1):158. Published 2019 Jul 19. doi:10.1186/s12903-019-0853-y |
| Ogawa H, Kurosaka K, Sato A, Hirasawa N, Matsubara M, Tsukada S. Does An Augmented Reality-based Portable Navigation System Improve the Accuracy of Acetabular Component Orientation During THA? A Randomized Controlled Trial. Clin Orthop Relat Res. 2020;478(5):935-943. doi:10.1097/CORR.0000000000001083 |
| Pietruski P, Majak M, Światek-Najwer E, et al. Supporting mandibular resection with intraoperative navigation utilizing augmented reality technology - A proof of concept study. J Craniomaxillofac Surg. 2019;47(6):854-859. doi:10.1016/j.jcms.2019.03.004 |
| Borgmann H, Rodríguez Socarrás M, Salem J, et al. Feasibility and safety of augmented reality-assisted urological surgery using smartglass. World J Urol. 2017;35(6):967-972. doi:10.1007/s00345-016-1956-6 |
| Shen J, Zemiti N, Taoum C, et al. Transrectal ultrasound image-based real-time augmented reality guidance in robot-assisted laparoscopic rectal surgery: a proof-of-concept study. Int J Comput Assist Radiol Surg. 2020;15(3):531-543. doi:10.1007/s11548-019-02100-2 |
| Elmi-Terander A, Burström G, Nachabe R, et al. Pedicle Screw Placement Using Augmented Reality Surgical Navigation With Intraoperative 3D Imaging: A First In-Human Prospective Cohort Study. Spine (Phila Pa 1976). 2019;44(7):517-525. doi:10.1097/BRS.0000000000002876 |
| Aguilera-Canon, M. C., Wainwright, T., Yang, X., & Nait-Charif, H. . Mixed Reality-Based Simulator for Training on Imageless Navigation Skills in Total Hip Replacement Procedures. In International Conference on E-Learning and Games (2018, June) (pp. 30-34). Springer, Cham. |
| Bradley D, Willson T, Chang JB, et al. Intraoperative Three-dimensional Virtual Reality and Computed Tomographic Guidance in Temporomandibular Joint Arthroplasty of Syndromic Craniofacial Dysostoses. Plast Reconstr Surg Glob Open. 2019;7(9):e2388. Published 2019 Sep 10. doi:10.1097/GOX.0000000000002388 |
| Buchs NC, Volonte F, Pugin F, et al. Augmented environments for the targeting of hepatic lesions during image-guided robotic liver surgery. J Surg Res. 2013;184(2):825-831. doi:10.1016/j.jss.2013.04.032 |
| Bjerrum F, Sorensen JL, Konge L, et al. Procedural specificity in laparoscopic simulator training: protocol for a randomised educational superiority trial. BMC Med Educ. 2014;14:215. Published 2014 Oct 10. doi:10.1186/1472-6920-14-215 |
| Rassweiler JJ, Müller M, Fangerau M, et al. iPad-assisted percutaneous access to the kidney using marker-based navigation: initial clinical experience. Eur Urol. 2012;61(3):628-631. doi:10.1016/j.eururo.2011.12.024 |
| Cohen, D., Mayer, E., Chen, D., Anstee, A., Vale, J., Yang, G. Z., & Darzi, A.. Augmented reality image guidance in minimally invasive prostatectomy. In International Workshop on Prostate Cancer Imaging (2010, September) (pp. 101-110). Springer, Berlin, Heidelberg. |
| Chessa, F., Schiavina, R., Bianchi, L., Marcelli, E., Diciotti, S., Lodi, S., ... & Brunocilla, E.. Three dimensional model of the prostate and augmented reality robot assisted radical prostatectomy: A randomized controlled study to evaluate intraoperative and pathologic outcomes. European Urology Open Science, (2020) 19, e1774-e1775. |
| Porpiglia, F., Checcucci, E., Amparore, D., Peretti, D., Piramide, F., De Cillis, S., ... & Fiori, C. . 3D mixed reality guidance for percutaneous puncture during kidney stones surgical treatment. European Urology Open Science,(2020) 19, e2331. |
| Ma CY, Zhou YL, Li HL, et al. Zhonghua Yi Xue Za Zhi. 2019;99(4):279-283. doi:10.3760/cma.j.issn.0376-2491.2019.04.008 |
| Gadjiev NK, Pogosyan RR, Ostanin MA, Petrov SB, Semenyakin IV. Urologiia. 2020;(5):37-40 |
| Souzaki R, Ieiri S, Uemura M, et al. An augmented reality navigation system for pediatric oncologic surgery based on preoperative CT and MRI images. J Pediatr Surg. 2013;48(12):2479-2483. doi:10.1016/j.jpedsurg.2013.08.025 |
| Schneider C, Thompson S, Totz J, et al. Comparison of manual and semi-automatic registration in augmented reality image-guided liver surgery: a clinical feasibility study. Surg Endosc. 2020;34(10):4702-4711. doi:10.1007/s00464-020-07807-x |
| Singla R, Edgcumbe P, Pratt P, Nguan C, Rohling R. Intra-operative ultrasound-based augmented reality guidance for laparoscopic surgery. Healthc Technol Lett. 2017;4(5):204-209. Published 2017 Sep 11. doi:10.1049/htl.2017.0063 |
| Adam, A., Robison, J., Lu, J., Jose, R., Badran, N., Vivas-Buitrago, T., ... & Huovinen, J. (2017, June). Abstracts from Hydrocephalus 2016. In Fluids and Barriers of the CNS (Vol. 14, No. 1, pp. 1-21). BioMed Central. |
| Brebant V, Heine N, Lamby P, et al. Augmented reality of indocyanine green fluorescence in simplified lymphovenous anastomosis in lymphatic surgery. Clin Hemorheol Microcirc. 2019;73(1):125-133. doi:10.3233/CH-199220 |
| E. L. Wisotzky et al., "Interactive and Multimodal-based Augmented Reality for Remote Assistance using a Digital Surgical Microscope," 2019 IEEE Conference on Virtual Reality and 3D User Interfaces (VR), 2019, pp. 1477-1484, doi: 10.1109/VR.2019.8797682. |
| Zeng B, Meng F, Ding H, Wang G. A surgical robot with augmented reality visualization for stereoelectroencephalography electrode implantation. Int J Comput Assist Radiol Surg. 2017;12(8):1355-1368. doi:10.1007/s11548-017-1634-1 |
| **Patient Pain** |
| Chan JJI, Yeam CT, Kee HM, et al. The use of pre-operative virtual reality to reduce anxiety in women undergoing gynecological surgeries: a prospective cohort study. BMC Anesthesiol. 2020;20(1):261. Published 2020 Oct 9. doi:10.1186/s12871-020-01177-6 |
| Ryu JH, Park SJ, Park JW, et al. Randomized clinical trial of immersive virtual reality tour of the operating theatre in children before anaesthesia. Br J Surg. 2017;104(12):1628-1633. doi:10.1002/bjs.10684 |
| Yang JH, Ryu JJ, Nam E, Lee HS, Lee JK. Effects of Preoperative Virtual Reality Magnetic Resonance Imaging on Preoperative Anxiety in Patients Undergoing Arthroscopic Knee Surgery: A Randomized Controlled Study. Arthroscopy. 2019;35(8):2394-2399. doi:10.1016/j.arthro.2019.02.037 |
| Bekelis K, Calnan D, Simmons N, MacKenzie TA, Kakoulides G. Effect of an Immersive Preoperative Virtual Reality Experience on Patient Reported Outcomes: A Randomized Controlled Trial. Ann Surg. 2017;265(6):1068-1073. doi:10.1097/SLA.0000000000002094 |
| Ganry L, Hersant B, Sidahmed-Mezi M, Dhonneur G, Meningaud JP. Using virtual reality to control preoperative anxiety in ambulatory surgery patients: A pilot study in maxillofacial and plastic surgery. J Stomatol Oral Maxillofac Surg. 2018;119(4):257-261. doi:10.1016/j.jormas.2017.12.010 |
| Noben L, Goossens SMTA, Truijens SEM, et al. A Virtual Reality Video to Improve Information Provision and Reduce Anxiety Before Cesarean Delivery: Randomized Controlled Trial. JMIR Ment Health. 2019;6(12):e15872. Published 2019 Dec 18. doi:10.2196/15872 |
| Ong TL, Ruppert MM, Akbar M, et al. Improving the Intensive Care Patient Experience With Virtual Reality-A Feasibility Study. Crit Care Explor. 2020;2(6):e0122. Published 2020 Jun 8. doi:10.1097/CCE.0000000000000122 |
| Eijlers R, Dierckx B, Staals LM, et al. Virtual reality exposure before elective day care surgery to reduce anxiety and pain in children: A randomised controlled trial. Eur J Anaesthesiol. 2019;36(10):728-737. doi:10.1097/EJA.0000000000001059 |
| Jung MJ, Libaw JS, Ma K, Whitlock EL, Feiner JR, Sinskey JL. Pediatric Distraction on Induction of Anesthesia With Virtual Reality and Perioperative Anxiolysis: A Randomized Controlled Trial. Anesth Analg. 2021;132(3):798-806. doi:10.1213/ANE.0000000000005004 |
| Mosso-Vázquez JL, Gao K, Wiederhold BK, Wiederhold MD. Virtual reality for pain management in cardiac surgery. Cyberpsychol Behav Soc Netw. 2014;17(6):371-378. doi:10.1089/cyber.2014.0198 |
| **Patient Anxiety** |
| Ong TL, Ruppert MM, Akbar M, et al. Improving the Intensive Care Patient Experience With Virtual Reality-A Feasibility Study. Crit Care Explor. 2020;2(6):e0122. Published 2020 Jun 8. doi:10.1097/CCE.0000000000000122 |
| Eijlers R, Dierckx B, Staals LM, et al. Virtual reality exposure before elective day care surgery to reduce anxiety and pain in children: A randomised controlled trial. Eur J Anaesthesiol. 2019;36(10):728-737. doi:10.1097/EJA.0000000000001059 |
| Faruki A, Nguyen T, Proeschel S, et al. Virtual reality as an adjunct to anesthesia in the operating room. Trials. 2019;20(1):782. Published 2019 Dec 27. doi:10.1186/s13063-019-3922-2 |
| Huang MY, Scharf S, Chan PY. Effects of immersive virtual reality therapy on intravenous patient-controlled sedation during orthopaedic surgery under regional anesthesia: A randomized controlled trial. PLoS One. 2020;15(2):e0229320. Published 2020 Feb 24. doi:10.1371/journal.pone.0229320 |
| Moon JY, Shin J, Chung J, Ji SH, Ro S, Kim WH. Virtual Reality Distraction during Endoscopic Urologic Surgery under Spinal Anesthesia: A Randomized Controlled Trial. J Clin Med. 2018;8(1):2. Published 2018 Dec 20. doi:10.3390/jcm8010002 |
| Kim, W. H., Ji, S. H., Moon, J. Y., & Choi, J. Y.. SEDATION USING IMMERSIVE VIRTUAL REALITY DURING UROLOGIC SURGERY UNDER SPINAL ANESTHESIA: A RANDOMIZED CONTROLLED TRIAL. In ANESTHESIA AND ANALGESIA (2018, April) (Vol. 126, No. 4, pp. 760-760). TWO COMMERCE SQ, 2001 MARKET ST, PHILADELPHIA, PA 19103 USA: LIPPINCOTT WILLIAMS & WILKINS. |
| Steele E, Grimmer K, Thomas B, Mulley B, Fulton I, Hoffman H. Virtual reality as a pediatric pain modulation technique: a case study. Cyberpsychol Behav. 2003;6(6):633-638. doi:10.1089/109493103322725405 |
| Esumi R, Yokochi A, Shimaoka M, Kawamoto E. Virtual reality as a non-pharmacologic analgesic for fasciotomy wound infections in acute compartment syndrome: a case report. J Med Case Rep. 2020;14(1):46. Published 2020 Apr 14. doi:10.1186/s13256-020-02370-4 |
| Haisley KR, Straw OJ, Müller DT, et al. Feasibility of implementing a virtual reality program as an adjuvant tool for peri-operative pain control; Results of a randomized controlled trial in minimally invasive foregut surgery. Complement Ther Med. 2020;49:102356. doi:10.1016/j.ctim.2020.102356 |
| Cacau Lde A, Oliveira GU, Maynard LG, et al. The use of the virtual reality as intervention tool in the postoperative of cardiac surgery. Rev Bras Cir Cardiovasc. 2013;28(2):281-289. doi:10.5935/1678-9741.20130039 |
| Gianola S, Stucovitz E, Castellini G, et al. Effects of early virtual reality-based rehabilitation in patients with total knee arthroplasty: A randomized controlled trial. Medicine (Baltimore). 2020;99(7):e19136. doi:10.1097/MD.0000000000019136 |
| **Surgical Training** |
| CARTER BN. The fruition of Halsted's concept of surgical training. Surgery. 1952 Sep;32(3):518-27. PMID: 12984268 |
| Al Janabi HF, Aydin A, Palaneer S, et al. Effectiveness of the HoloLens mixed-reality headset in minimally invasive surgery: a simulation-based feasibility study. Surg Endosc. 2020;34(3):1143-1149. doi:10.1007/s00464-019-06862-3 |
| Larsen, C., Soerensen, J., Grantcharov, T., Dalsgaard, T., Schouenborg, L., Ottosen, C., ... & Ottesen, B. (2009). O507 Impact of virtual reality training in laparoscopic gynaecology. International Journal of Gynecology & Obstetrics, 107, S237-S237. |
| Larsen CR, Soerensen JL, Grantcharov TP, et al. Effect of virtual reality training on laparoscopic surgery: randomised controlled trial [published correction appears in BMJ. 2009;338. doi: 10.1136/bmj.b2074]. BMJ. 2009;338:b1802. Published 2009 May 14. doi:10.1136/bmj.b1802 |
| Jokinen E, Mikkola TS, Härkki P. Simulator training and residents' first laparoscopic hysterectomy: a randomized controlled trial. Surg Endosc. 2020;34(11):4874-4882. doi:10.1007/s00464-019-07270-3 |
| Ahlberg G, Enochsson L, Gallagher AG, et al. Proficiency-based virtual reality training significantly reduces the error rate for residents during their first 10 laparoscopic cholecystectomies. Am J Surg. 2007;193(6):797-804. doi:10.1016/j.amjsurg.2006.06.050 |
| Aggarwal R, Tully A, Grantcharov T, et al. Virtual reality simulation training can improve technical skills during laparoscopic salpingectomy for ectopic pregnancy. BJOG. 2006;113(12):1382-1387. doi:10.1111/j.1471-0528.2006.01148.x |
| Sumitani D, Egi H, Tokunaga M, et al. Virtual reality training followed by box training improves the laparoscopic skills of novice surgeons. Minim Invasive Ther Allied Technol. 2013;22(3):150-156. doi:10.3109/13645706.2012.721377 |
| Waterman BR, Martin KD, Cameron KL, Owens BD, Belmont PJ Jr. Simulation Training Improves Surgical Proficiency and Safety During Diagnostic Shoulder Arthroscopy Performed by Residents. Orthopedics. 2016;39(3):e479-e485. doi:10.3928/01477447-20160427-02 |
| Hooper J, Tsiridis E, Feng JE, et al. Virtual Reality Simulation Facilitates Resident Training in Total Hip Arthroplasty: A Randomized Controlled Trial. J Arthroplasty. 2019;34(10):2278-2283. doi:10.1016/j.arth.2019.04.002 |
| Logishetty K, Rudran B, Cobb JP. Virtual reality training improves trainee performance in total hip arthroplasty: a randomized controlled trial. Bone Joint J. 2019;101-B(12):1585-1592. doi:10.1302/0301-620X.101B12.BJJ-2019-0643.R1 |
| Logishetty K, Gofton WT, Rudran B, Beaulé PE, Cobb JP. Fully Immersive Virtual Reality for Total Hip Arthroplasty: Objective Measurement of Skills and Transfer of Visuospatial Performance After a Competency-Based Simulation Curriculum. J Bone Joint Surg Am. 2020;102(6):e27. doi:10.2106/JBJS.19.00629 |
| Xin B, Huang X, Wan W, et al. The efficacy of immersive virtual reality surgical simulator training for pedicle screw placement: a randomized double-blind controlled trial. Int Orthop. 2020;44(5):927-934. doi:10.1007/s00264-020-04488-y |
| Hou Y, Shi J, Lin Y, Chen H, Yuan W. Virtual surgery simulation versus traditional approaches in training of residents in cervical pedicle screw placement. Arch Orthop Trauma Surg. 2018;138(6):777-782. doi:10.1007/s00402-018-2906-0 |
| Mariani A, Pellegrini E, Enayati N, Kazanzides P, Vidotto M, De Momi E. Design and Evaluation of a Performance-based Adaptive Curriculum for Robotic Surgical Training: a Pilot Study. Annu Int Conf IEEE Eng Med Biol Soc. 2018;2018:2162-2165. doi:10.1109/EMBC.2018.8512728 |
| Saratzis A, Calderbank T, Sidloff D, Bown MJ, Davies RS. Role of Simulation in Endovascular Aneurysm Repair (EVAR) Training: A Preliminary Study. Eur J Vasc Endovasc Surg. 2017;53(2):193-198. doi:10.1016/j.ejvs.2016.11.016 |
| Varshney R, Frenkiel S, Nguyen LH, et al. Development of the McGill simulator for endoscopic sinus surgery: a new high-fidelity virtual reality simulator for endoscopic sinus surgery. Am J Rhinol Allergy. 2014;28(4):330-334. doi:10.2500/ajra.2014.28.4046 |
| Kowalewski KF, Garrow CR, Proctor T, et al. LapTrain: multi-modality training curriculum for laparoscopic cholecystectomy-results of a randomized controlled trial. Surg Endosc. 2018;32(9):3830-3838. doi:10.1007/s00464-018-6110-7 |
| Thomsen AS, Bach-Holm D, Kjærbo H, et al. Operating Room Performance Improves after Proficiency-Based Virtual Reality Cataract Surgery Training. Ophthalmology. 2017;124(4):524-531. doi:10.1016/j.ophtha.2016.11.015 |
| Feudner EM, Engel C, Neuhann IM, Petermeier K, Bartz-Schmidt KU, Szurman P. Virtual reality training improves wet-lab performance of capsulorhexis: results of a randomized, controlled study. Graefes Arch Clin Exp Ophthalmol. 2009;247(7):955-963. doi:10.1007/s00417-008-1029-7 |
| McCannel CA, Reed DC, Goldman DR. Ophthalmic surgery simulator training improves resident performance of capsulorhexis in the operating room. Ophthalmology. 2013;120(12):2456-2461. doi:10.1016/j.ophtha.2013.05.003 |
| Ferris JD, Donachie PH, Johnston RL, Barnes B, Olaitan M, Sparrow JM. Royal College of Ophthalmologists' National Ophthalmology Database study of cataract surgery: report 6. The impact of EyeSi virtual reality training on complications rates of cataract surgery performed by first and second year trainees. Br J Ophthalmol. 2020;104(3):324-329. doi:10.1136/bjophthalmol-2018-313817 |
| Kailavasan M, Berridge C, Athanasiadis G, et al. Design, implementation, and evaluation of a novel curriculum to teach transurethral resection of the prostate (TURP): a 3-year experience of urology simulation bootcamp course. World J Urol. 2020;38(11):2899-2906. doi:10.1007/s00345-020-03104-3 |
| Schulz GB, Grimm T, Kretschmer A, Stief CG, Jokisch F, Karl A. Benefits and Limitations of Transurethral Resection of the Prostate Training With a Novel Virtual Reality Simulator. Simul Healthc. 2020;15(1):14-20. doi:10.1097/SIH.0000000000000396 |
| Beyer-Berjot L, Pucher P, Patel V, et al. Colorectal surgery and enhanced recovery: Impact of a simulation-based care pathway training curriculum. J Visc Surg. 2017;154(5):313-320. doi:10.1016/j.jviscsurg.2017.02.003 |
| Gawęcki W, Węgrzyniak M, Mickiewicz P, Gawłowska MB, Talar M, Wierzbicka M. The Impact of Virtual Reality Training on the Quality of Real Antromastoidectomy Performance. J Clin Med. 2020;9(10):3197. Published 2020 Oct 2. doi:10.3390/jcm9103197 |
| Arora H, Uribe J, Ralph W, et al. Assessment of construct validity of the endoscopic sinus surgery simulator. Arch Otolaryngol Head Neck Surg. 2005;131(3):217-221. doi:10.1001/archotol.131.3.217 |
| Bittner JG 4th, Mellinger JD, Imam T, Schade RR, Macfadyen BV Jr. Face and construct validity of a computer-based virtual reality simulator for ERCP. Gastrointest Endosc. 2010;71(2):357-364. doi:10.1016/j.gie.2009.08.033 |
| Kuronen-Stewart C, Ahmed K, Aydin A, et al. Holmium Laser Enucleation of the Prostate: Simulation-Based Training Curriculum and Validation. Urology. 2015;86(3):639-646. doi:10.1016/j.urology.2015.06.008 |
| Whittaker G, Aydin A, Raison N, et al. Validation of the RobotiX Mentor Robotic Surgery Simulator. J Endourol. 2016;30(3):338-346. doi:10.1089/end.2015.0620 |
| Huang C, Cheng H, Bureau Y, Ladak HM, Agrawal SK. Automated Metrics in a Virtual-Reality Myringotomy Simulator: Development and Construct Validity. Otol Neurotol. 2018;39(7):e601-e608. doi:10.1097/MAO.0000000000001867 |
| Iwata N, Fujiwara M, Kodera Y, et al. Construct validity of the LapVR virtual-reality surgical simulator. Surg Endosc. 2011;25(2):423-428. doi:10.1007/s00464-010-1184-x |
| Wijn RP, Persoon MC, Schout BM, Martens EJ, Scherpbier AJ, Hendrikx AJ. Virtual reality laparoscopic nephrectomy simulator is lacking in construct validity. J Endourol. 2010;24(1):117-122. doi:10.1089/end.2009.0219 |
| van der Meijden OA, Broeders IA, Schijven MP. The SEP "robot": a valid virtual reality robotic simulator for the Da Vinci Surgical System?. Surg Technol Int. 2010;19:51-58. |
| Nayar SK, Musto L, Fernandes R, Bharathan R. Validation of a virtual reality laparoscopic appendicectomy simulator: a novel process using cognitive task analysis. Ir J Med Sci. 2019;188(3):963-971. doi:10.1007/s11845-018-1931-x |
| Whittaker G, Aydin A, Raveendran S, Dar F, Dasgupta P, Ahmed K. Validity assessment of a simulation module for robot-assisted thoracic lobectomy. Asian Cardiovasc Thorac Ann. 2019;27(1):23-29. doi:10.1177/0218492318813457 |
| Wynn G, Lykoudis P, Berlingieri P. Development and implementation of a virtual reality laparoscopic colorectal training curriculum. Am J Surg. 2018;216(3):610-617. doi:10.1016/j.amjsurg.2017.11.034 |
| Neary PC, Boyle E, Delaney CP, Senagore AJ, Keane FB, Gallagher AG. Construct validation of a novel hybrid virtual-reality simulator for training and assessing laparoscopic colectomy; results from the first course for experienced senior laparoscopic surgeons. Surg Endosc. 2008;22(10):2301-2309. doi:10.1007/s00464-008-9900-5 |
| Ayodeji ID, Schijven M, Jakimowicz J, Greve JW. Face validation of the Simbionix LAP Mentor virtual reality training module and its applicability in the surgical curriculum. Surg Endosc. 2007;21(9):1641-1649. doi:10.1007/s00464-007-9219-7 |
| Botden SM, Berlage JT, Schijven MP, Jakimowicz JJ. Face validity study of the ProMIS augmented reality laparoscopic suturing simulator. Surg Technol Int. 2008;17:26-32. |
| Hennessey IA, Hewett P. Virtual reality versus box laparoscopic simulators in trainee selection and aptitude testing. Surg Laparosc Endosc Percutan Tech. 2014;24(4):318-321. doi:10.1097/SLE.0b013e3182a2f05f |
| Nugent E, Shirilla N, Hafeez A, et al. Development and evaluation of a simulator-based laparoscopic training program for surgical novices. Surg Endosc. 2013;27(1):214-221. doi:10.1007/s00464-012-2423-0 |
| Meneghetti AT, Pachev G, Zheng B, Panton ON, Qayumi K. Objective assessment of laparoscopic skills: dual-task approach. Surg Innov. 2012;19(4):452-459. doi:10.1177/1553350611430673 |
| Schmidt MW, Kowalewski KF, Schmidt ML, et al. The Heidelberg VR Score: development and validation of a composite score for laparoscopic virtual reality training. Surg Endosc. 2019;33(7):2093-2103. doi:10.1007/s00464-018-6480-x |
| Kailavasan M, Berridge C, Athanasiadis G, et al. Design, implementation, and evaluation of a novel curriculum to teach transurethral resection of the prostate (TURP): a 3-year experience of urology simulation bootcamp course. World J Urol. 2020;38(11):2899-2906. doi:10.1007/s00345-020-03104-3 |
| Larcher A, Turri F, Bianchi L, et al. Virtual Reality Validation of the ERUS Simulation-based Training Programmes: Results from a High-volume Training Centre for Robot-assisted Surgery. Eur Urol. 2019;75(5):885-887. doi:10.1016/j.eururo.2019.02.008 |
| Burden C, Appleyard TL, Angouri J, Draycott TJ, McDermott L, Fox R. Implementation of laparoscopic virtual-reality simulation training in gynaecology: a mixed-methods design. Eur J Obstet Gynecol Reprod Biol. 2013;170(2):474-479. doi:10.1016/j.ejogrb.2013.07.003 |
| ROGISTER, F., SALMON, C., GHUYSEN, A., BONNET, P., CAMBY, S., LEFEBVRE, P., & POIRRIER, A. L.. Surgical Simulation in Temporal Bone Surgical Radio-Anatomy Learning. (2019) B-ENT. |
| Gasperin BDM, Zanirati T, Cavazzola LT. CAN VIRTUAL REALITY BE AS GOOD AS OPERATING ROOM TRAINING? EXPERIENCE FROM A RESIDENCY PROGRAM IN GENERAL SURGERY. Arq Bras Cir Dig. 2018;31(4):e1397. Published 2018 Dec 6. doi:10.1590/0102-672020180001e1397 |
| Maschuw K, Schlosser K, Kupietz E, Slater EP, Weyers P, Hassan I. Do soft skills predict surgical performance?: a single-center randomized controlled trial evaluating predictors of skill acquisition in virtual reality laparoscopy. World J Surg. 2011;35(3):480-486. doi:10.1007/s00268-010-0933-2 |
| Raison N, Gavazzi A, Abe T, Ahmed K, Dasgupta P. Virtually Competent: A Comparative Analysis of Virtual Reality and Dry-Lab Robotic Simulation Training. J Endourol. 2020;34(3):379-384. doi:10.1089/end.2019.0541 |
| Yudkowsky R, Luciano C, Banerjee P, et al. Practice on an augmented reality/haptic simulator and library of virtual brains improves residents' ability to perform a ventriculostomy. Simul Healthc. 2013;8(1):25-31. doi:10.1097/SIH.0b013e3182662c69 |
| Rhienmora, P., Gajananan, K., Haddawy, P., Suebnukarn, S., Dailey, M. N., Supataratarn, E., & Shrestha, P. (2010, February). Haptic augmented reality dental trainer with automatic performance assessment. In Proceedings of the 15th international conference on Intelligent user interfaces (pp. 425-426). |
| Shu, S. H., Su, E. L. M., Yeong, C. F., Sood, S., & Gandhi, A. (2017). Development of Virtual Reality Surgical Simulator with Online Database. Journal of Telecommunication, Electronic and Computer Engineering (JTEC), 9(3-9), 91-94. |
| Vasudevan, M. K., Isaac, J. H., Sadanand, V., & Muniyandi, M. (2020). Novel virtual reality based training system for fine motor skills: Towards developing a robotic surgery training system. The International Journal of Medical Robotics and Computer Assisted Surgery, 16(6), 1-14. |
| Pulijala Y, Ma M, Pears M, Peebles D, Ayoub A. Effectiveness of Immersive Virtual Reality in Surgical Training-A Randomized Control Trial. J Oral Maxillofac Surg. 2018;76(5):1065-1072. doi:10.1016/j.joms.2017.10.002 |
| Thomsen, A. S. S. S., Kiilgaard, J. F., De La Cour, M. D., & Konge, L. Investigating inter-procedural transfer of surgical skills using virtual-reality simulation. (2016) Investigative Ophthalmology & Visual Science, 57(12), 5827-5827. |
| Hudak SJ, Landt CL, Hernandez J, Soderdahl DW. External validation of a virtual reality transurethral resection of the prostate simulator. J Urol. 2010;184(5):2018-2022. doi:10.1016/j.juro.2010.06.141 |
| Jacobsen MF, Konge L, Bach-Holm D, et al. Correlation of virtual reality performance with real-life cataract surgery performance. J Cataract Refract Surg. 2019;45(9):1246-1251. doi:10.1016/j.jcrs.2019.04.007 |
| Raison N, Ahmed K, Fossati N, et al. Competency based training in robotic surgery: benchmark scores for virtual reality robotic simulation. BJU Int. 2017;119(5):804-811. doi:10.1111/bju.13710 |
| **Surgeon Confidence** |
| Chugh AJ, Pace JR, Singer J, et al. Use of a surgical rehearsal platform and improvement in aneurysm clipping measures: results of a prospective, randomized trial. J Neurosurg. 2017;126(3):838-844. doi:10.3171/2016.1.JNS152576 |
| Calatayud D, Arora S, Aggarwal R, et al. Warm-up in a virtual reality environment improves performance in the operating room. Ann Surg. 2010;251(6):1181-1185. doi:10.1097/SLA.0b013e3181deb630 |
| Deuchler S, Wagner C, Singh P, et al. Clinical Efficacy of Simulated Vitreoretinal Surgery to Prepare Surgeons for the Upcoming Intervention in the Operating Room. PLoS One. 2016;11(3):e0150690. Published 2016 Mar 10. doi:10.1371/journal.pone.0150690 |
| Kroft J, Ordon M, Po L, Zwingerman N, Lee JY, Pittini R. Can Surgical "Warm-Up" With Instructor Feedback Improve Operative Performance of Surgical Trainees?. J Minim Invasive Gynecol. 2015;22(6S):S17-S18. doi:10.1016/j.jmig.2015.08.057 |
| Lendvay TS, Brand TC, White L, et al. Virtual reality robotic surgery warm-up improves task performance in a dry laboratory environment: a prospective randomized controlled study. J Am Coll Surg. 2013;216(6):1181-1192. doi:10.1016/j.jamcollsurg.2013.02.012 |
| Coelho G, Defino HLA. The Role of Mixed Reality Simulation for Surgical Training in Spine: Phase 1 Validation. Spine (Phila Pa 1976). 2018;43(22):1609-1616. doi:10.1097/BRS.0000000000002856 |
| Ayodeji ID, Schijven M, Jakimowicz J, Greve JW. Face validation of the Simbionix LAP Mentor virtual reality training module and its applicability in the surgical curriculum. Surg Endosc. 2007;21(9):1641-1649. doi:10.1007/s00464-007-9219-7 |
